# Comparing Outcomes of Two Vascular Inflow Occlusion Techniques and Treatment without Vascular Occlusion during Major Hepatectomy in Patients with Hepatitis B-Related Hepatocellular Carcinoma

**DOI:** 10.1371/journal.pone.0107303

**Published:** 2014-09-09

**Authors:** Zhiping Huang, Peng Zhang, Haiqing Wang, Lunan Yan, Wentao Wang

**Affiliations:** 1 Department of hepatopancreatobiliary Surgery, Yongchuan Hospital, Chongqing Medical University, Yongchuan, Chongqing, China; 2 Department of Liver Surgery, Liver Transplantation Center, West China Hospital of Sichuan University, Chengdu, Sichuan Province, China; University Hospital Heidelberg, Germany

## Abstract

**Background:**

Significant hemorrhage together with blood transfusion has negative impact on postoperative morbidity, mortality, and long-term survival of liver resection. Various techniques of vascular occlusion have been developed to reduce intraoperative blood loss. The objective of this study was to compare the outcomes of Pringle maneuver, hemi-hepatic vascular occlusion, and treatment without vascular occlusion used during liver resection.

**Method:**

Data of 574 patients with Hepatitis B virus (HBV)-related hepatocellular carcinoma (HCC), who underwent major hepatectomy between January 2009 to March 2013 by Pringle maneuver (N = 158), hemi-hepatic vascular inflow occlusion (N = 216), or without any vascular occlusion (N = 200), were included in this retrospective study. Perioperative blood transfusion, intraoperative blood loss, and postoperative liver function, and surgical complications were analyzed and compared between the three groups.

**Result:**

There were no significant difference observed in postoperative bilirubin, liver enzyme, and albumin levels between three groups (P>0.05). 5 patients (2.5%) in no occlusion group, 2 (1.3%) in Pringle group, and 8 (3.7%) in hemi-hepatic group had liver failure; but, there were no differences (P>0.05). The overall postoperative complications rate between three groups did not reach significant differences (33.5% vs 34.2% vs 42.6%, respectively; P>0.05). However, significant differences in intraoperative blood loss between no occlusion group (638.2±426.8 ml) and Pringle group (518.0±451.0 ml) or hemi-hepatic group (513.0±366.7 ml) (P<0.01).

**Conclusion:**

Although there were no differences found between three groups regarding postoperative complications rate, no vascular occlusion group had more blood loss than the other two groups during liver resection.

## Introduction

Hepatocellular Carcinoma (HCC) is one of the most prevalent and deadly cancers [1]; it is the sixth most common cancer worldwide with 749,000 new cases yearly, and the third leading cause of cancer deaths with 692,000 deaths yearly. In China, a high incidence of HCC with cirrhosis is largely due to hepatitis B infections, which is in marked contrast to the West, where the main causes of cirrhosis are hepatitis C or alcohol related[2]–[4]. Currently, there are various effective treatments available for HCC such as liver resection, liver transplantation, radiofrequency ablation, and transcatheter hepatic arterial chemoembolization. However, partial hepatectomy is still the most commonly used technique [5], and Barcelona Clinic Liver Cancer (BCLC) staging system recommends it as the primary therapeutic method for patients with early stage HCC [6].

Liver resection has experienced more than 100 years of development as the primary treatment for HCC. To the end of 19th century, the animal experiments had shown the feasibility of liver parenchyma incision. William keen (1899) was considered as the first American surgeon to perform liver resection; he reported three successful surgical cases. However, the perioperative mortality (70%–90%) of liver resection was very high during that period [7]. One of main reasons was that intraoperative blood loss in the surgery could not be effectively controlled. Significant hemorrhage together with blood transfusion increases postoperative morbidity and mortality of hepatic resection [8]–[10]. Various techniques of vascular occlusion have been developed to reduce intraoperative blood loss.

The Pringle maneuver, a technique of transient hepatic vascular inflow occlusion, was described by J.H.Pringle, a British surgeon in 1908 [11]. It is performed encircling the hepatoduodenal ligament with a tape and then applying a tourniquet or vascular clamp until the hepatic arterial pulse disappears distally. The validity of this technique in reducing hemorrhage in liver resection had been proved by Man et al [12]. However, the Pringle maneuver can induce ischemia-reperfusion injury to the remnant liver [13], [14], and some surgeons even claimed that the Pringle maneuver should be avoided in the partial hepatectomy because of its induction of tumor recurrence and worse prognosis [15], [16].

To avoid ischemia-reperfusion injury to the remnant liver, Makuuchi [17] et al proposed a hemi-hepatic inflow vascular occlusion technique, which could only block the blood supply of right or left hemi-hepatic at tumor location, allowing normal blood supply at contralateral hemi-liver. The advantage of this technique is especially seen in patients with cancer who need right or left hemi-liver resection, because no liver tissues would subject to ischemia-reperfusion. However, the adverse effect of hemi-hepatic vascular occlusion includes more bleeding from the raw surface of the other hemi-liver. Liver surgeons should be experienced in dissecting and lowering hilar plate to avoid any injury to bile ducts and vessels. Although the Pringle maneuver and hemi-hepatic vascular occlusion are the most commonly used techniques in clinical practice, no consensus has still arrived among surgeons on choosing an optimal method during liver.

This retrospective cohort study was designed to compare the outcomes of Pringle maneuver, hemi-hepatic vascular occlusion, and treatment without vascular occlusion used during liver resection in patients with HCC.

## Patients and Methods

### Trail design

This study was approved by Institutional Review Board of the West China Hospital of Sichuan University. Although written or verbal informed consent given by participants could not be obtained, patient records/information was anonymized and de-identified. All the data were collected retrospectively from the HCC database of the hospital.

From January 2009 to March 2013, 574 patients with Hepatitis B virus (HBV)-related HCC who underwent major hepatectomy [18] (defined as the resection of liver, at least, three Couianud’s segments), formed this study set. The diagnosis of HCC was based on American Association for the Study of Liver Diseases (AASLD) and European Association for the Study of the Liver (EASL) guidelines [1], [19], [20] and proved by cytological/histological after resection. All patients were operated only when Child-Turcotte-Pugh (CTP) score was A, or CTP class A was reached by the treatment that adopted for patients with CTP class B. Patients who met the following criteria were excluded from the study: (1) diagnosis was not HCC; (2) no requirement for major hepatectomy; (3) a history of previous partial hepatectomy; and (4) having major concomitant surgery including adrenal resection, esophageal devascularization, and gastrostomy. All surgical procedures were guided by the same team of liver surgeons who had experience in liver resections. The main endpoints were the intraoperative blood loss, postoperative liver function, postoperative morbidity and mortality rate.

All patients were divided into 3 groups according to the technique of vascular occlusion used in hepatic resection: no vascular occlusion, Pringle maneuver, and hemi-hepatic vascular inflow occlusion groups.

### Preoperative examination

All patients had a chest x-ray, electrocardiogram, ultrasonography, and contrast computed tomography or magnetic resonance imaging of abdomen. Laboratory blood tests included blood routine, antigen of hepatitis B (HBsAg), antibody of hepatitis C (HCVAb), aspartate aminotransferase (AST), alanine aminotransferase (ALT), serum total bilirubin (TB), alpha-fetoprotein (AFP), and prothrombin time (PT). The Anesthesia grade [21] (American Society of Anesthesiologists), Charlson index [22] (used to quantify preoperative comorbidities), and CTP score were determined.

### Surgical procedure

Right subcostal oblique incision was chosen to perform surgery. When the abdominal cavity was opened, intraoperative ultrasonography was used routinely to check the extent of tumor and its position and to assess whether there were any extra-hepatic metastases. The liver was mobilized as soon as the resectability of the tumor was determined. After that, the technique of vascular occlusion was selected or not, according to the surgeon’s preference and patient’s intraoperative condition.

The Pringle maneuver was performed by encircling the hepatoduodenal ligament with a catheter and then applying a vascular clamp until the hepatic arterial pulse disappeared distally. Tightening or loosening the catheter resulted in blocking or not blocking the vascular inflow to the liver. In this method, intermittent vascular occlusion was applied. The circulation of blocking and not blocking vascular inflow was 15/5 min.

**Figure 1:**
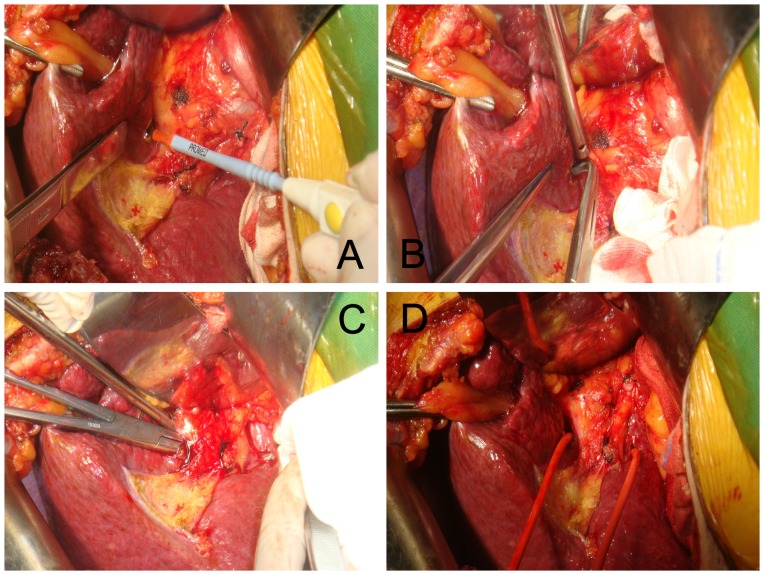
Simple hemi-occlusion. **A:** On the visceral envelope overlying the confluence, a small hole was made using a sharp blade; **B:** A right-angle forceps was inserted to gently mobilize the liver parenchyma outside Glisson’s sheath; **C:** The right-angle forceps should mobilize in the liver parenchyma towards the caudate lobe; **D:** A catheter was introduced.

A special technique called lowering the liver hilar plate, which was put forward by Hepp and Couinaud in 1956, was adopted [23], when performing hemi-hepatic vascular occlusion. However, this technique had a potential risk to injure the bile ducts and vessels. Procedures of hemi-hepatic vascular occlusion from our center had been described in previous published works [24], [25] (Figure 1).

**Figure 2:**
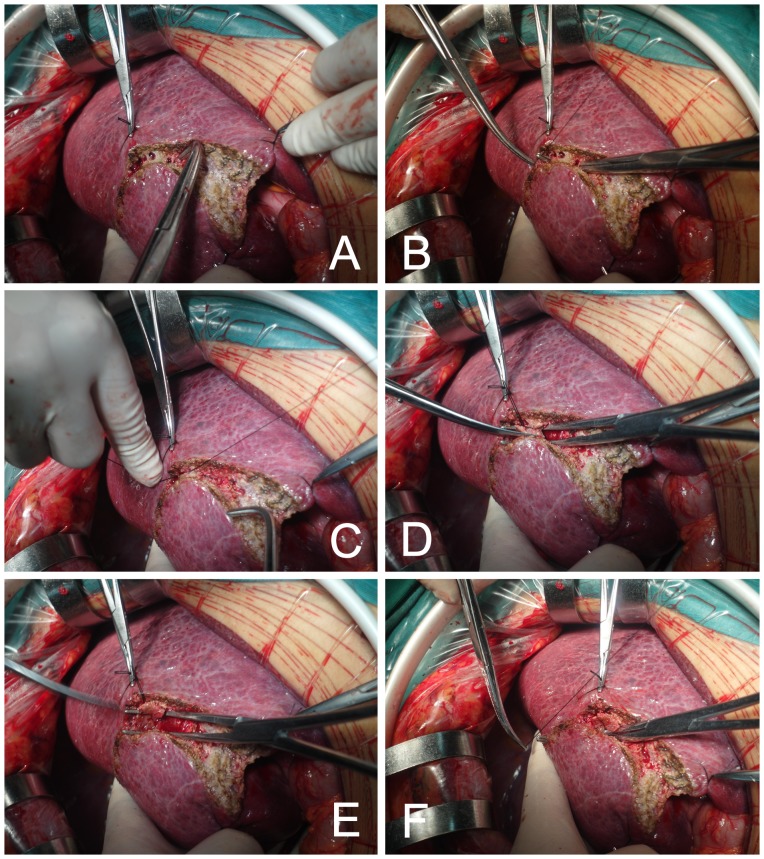
Hooking with ligation. A: The resection line was marked by electrocautery on the hepatic surface, B: The liver was dissected by rightangle forceps hooking the hepatic tissue; C, D, and E: Cannular structures were ligated and cut; F: Right-angle forceps hooked the hepatic tissue forward one by one.

For liver parenchymal division, hooking with ligation or Cavitron ultrasonic surgical aspirator (CUSA) was used. Hooking with ligation is a simple and effective technique for liver resection, introduced by Yan in 1994 [24], [25] (Figure 2). When no vascular occlusion or hemi-occlusion technique was used in the liver surgery, CUSA was selected for liver parenchymal transection. However, when the Pringle maneuver was used in the surgery, hooking with ligation was chosen, because only a short time (always less than 30 min) was needed for liver resection.

### Postoperative evaluation

The measurements of blood loss were made before, during, and after liver resection. The volume of blood loss was measured from the weight of the soaked gauze and blood collected from the containers of the suction apparatus and ultrasonic dissector. The volume of irrigation fluid was deduced accordingly. Generally speaking, whole blood was not transfused unless the hematocrit value became less than 30% during surgery. All patients were carefully observed after operation. Admission at the intensive care unit (ICU) was determined by patients’ intraoperative condition. Routine tests of liver function were performed after operation. Mortality (defined as death within 90 days after surgery or death during the same hospital admission of surgery), morbidity, postoperative hospitalization duration, and ICU stay were record in detail.

### Statistical analysis

Statistical analysis was performed using SPSS (Version 17.0). The results of continuous variables were expressed as means ± standard deviation or median with interquartile range. The variables were compared using one-way analysis of variance. Non-parametric variables used the Mann-Whitney U test or Kruskal-wallis test. Chi-square test was used to compare categorical variables. P<0.05 was considered statistically significant.

## Result

In this retrospective study, data of 574 patients with HBV-related HCC were analyzed. The preoperative demographic and baseline data are described in Table 1. There were no significant differences between three groups regarding age, sex, Charlson score, portal hypertension (defined as esophageal varices detected by endoscopy or a splenomegaly (major diameter >12 cm) with a platelet count <100 000/mm3 according to the BCLC group criteria [19], [20]), anesthesia grade, and laboratory blood tests related to liver function. However, most patients in three groups had different degree of liver cirrhosis during liver resection.

**Table 1:** Preoperative characteristic of patients.

	No occlusion group (n = 200)	The Pringle group (n = 158)	The hemi-hepatic group (n = 126)	P value
**Age(years)**	50.4±12.6	48.5±12.0	49.3±12.8	0.358
**Sex(M/F)**	175/25	129/29	187/29	0.253
**Charlson score**	1(1–3)	1(1–3)	1(1–3)	0.573
**Median(IQR)**				
**Portal hypertension**	24(12.0%)	31(19.6%)	31(14.4%)	0.127
**Anesthesia grade**	169/31	137/21	187/29	0.783
**(I+II/III+IV)**				
**HBsAg(+)**	155(77.5%)	134(84.8%)	169(78.2%)	0.179
**ALT (u/l)**	56.8±52.6	55.9±45.8	58.0±49.4	0.918
**AST (u/l)**	63.1±70.6	62.8±50.6	57.5±47.7	0.545
**Albumin(g/l)**	40.6±4.6	39.4±5.3	39.9±4.6	0.063
**Hemoglobin(g/l)**	138.5±20.0	138.5±22.2	140.5±20.0	0.516
**Plate(10x9/l)**	167.5±80.6	163.9±79.8	175.3±83.6	0.378
**Leukocyte(10x9/l)**	6.5±2.4	6.1±2.7	6.3±2.2	0.301

**IQR:** interquartile range; **ALT:** alanine aminotransferase; **AST:** aspartate aminotransferase;

**HBsAg:** antigen of hepatitis B.

### Result of occlusion time, intraoperative blood loss, blood transfusion requirement

The intraoperative data are listed in Table 2. 141 patients (24.6%) had perioperative blood transfusion. Occlusion time in the Pringle group was 41.9±12.3 min vs 44.3±18.7 min in the hemi-hepatic group (P>0.05).

**Table 2:** Intraoperative data.

	No occlusion group (n = 200)	The Pringle group (n = 158)	The hemi-hepatic group (n = 216)	P value
**Types of resection**				
**3 segments of liver**	91(45.5%)	68(43.0%)	114(52.8%)	0.136
**4 segments of liver**	101(50.5%)	76(48.1%)	91(42.1%)	0.213
**5 segments of liver**	8(4.0%)	14(9.9%)	11(5.1%)	0.127
**Occlusion time(min)**	0	41.9±12.3	44.3±18.7	0.150
**Intraoperative**	638.2±426.8	518.0±451.0	513.0±366.7	0.03
**Blood loss(ml)**				I vs II = 0.906
				I vs III = 0.001
				II vs III = 0.01
**Blood transfusion**	45/155(22.5%)	42/166(26.6%)	54/162(25.0%)	0.661
**YES/NO**				

**I:** Hemi-hepatic group, **II**: Pringle group, **III:** No occlusion group.

There were significant differences between three groups in intraoperative blood loss (P<0.01). The median blood loss in the Pringle group was 518.0±451.0 ml vs 513.0±366.7 ml in the hemi-hepatic group (P>0.05). There were significant differences between no occlusion group (638.2±426.8 ml) and Pringle or hemi-hepatic groups in intraoperative blood loss (P<0.01). There were also no difference in blood transfusion requirements (P>0.05).

About 45.5% had three segments of liver resection in no occlusion group vs 43.0% in Pringle group and 52.8% in hemi-hepatic group (P>0.05). The percentage of patients who had four segments of liver resection included 50.5%, 48.1%, and 42.1%, respectively in no occlusion group, Pringle group, and hemi-hepatic group (P>0.05). There were also no significant differences between three groups of patients who had five segments of liver resection (4.0% vs 9.9% vs 5.1%, respectively, P>0.05).

### Result of postoperative liver function

The postoperative liver function data are listed in Table 3. Serum total bilirubin and liver enzymes were increased in three groups on days 1, 3, and 5. The elevated bilirubin and liver enzymes had a tendency to return to the baseline value on 5th postoperative day. However, there were no significant difference observed between three groups in bilirubin, liver enzymes, and albumin (P>0.05).

**Table 3:** The postoperative liver function data.

	No occlusion group (n = 200)	The Pringle group (n = 158)	The hemi-hepatic group (n = 216)	P value
**Day 1**				
** TBIL(umol/l)**	28.0±16.4	28.2±15.2	30.8±20.0	0.209
** ALT(u/l)**	413.9±415.8	409.7±319.8	432.5±574.5	0.873
** AST(u/l)**	392.2±389.7	417.5±390.5	438.7±623.2	0.627
** Albumin(g/l)**	28.4±5.1	28.3±5.4	28.8±5.6	0.560
**Day 3**				
** TBIL(umol/l)**	33.3±20.4	33.5±19.8	37.1±31.5	0.229
** ALT(u/l)**	282.1±262.0	296.9±243.2	281.8±266.9	0.827
** AST(u/l)**	139.7±123.7	151.6±140.6	147.0±129.0	0.681
** Albumin(g/l)**	31.7±4.1	31.2±4.6	31.4±5.1	0.578
**Day 5**				
** TBIL(umol/l)**	28.0±24.1	28.0±20.8	32.1±32.0	0.196
** ALT(u/l)**	127.7±106.1	133.2±104.4	129.4±111.8	0.885
** AST(u/l)**	56.4±37.3	60.8±40.8	66.4±68.3	0.141
** Albumin(g/l)**	34.1±4.8	34.5±5.6	34.8±5.0	0.422

**TBIL:** total serum bilirubin; **ALT:** alanine aminotransferase; **AST:** aspartate aminotransferase;

Five patients (2.5%) in no occlusion group, 2 (1.3%) in Pringle group, and 8 (3.7%) in hemi-hepatic group had liver failure (defined as prothrombin time<50% and serum bilirubin level>50 µmol/L on the on day 5 after liver resection [28]) and they did not reach significant difference (P>0.05, Table 4).

**Table 4:** Postoperative data.

	No occlusion group (n = 200)	The Pringle group (n = 158)	The hemi-hepatic group (n = 216)	P value
**The days of staying** **in ICU (IQR)**	0(0–1)	0(0–1)	0(0–1)	0.604
**Hospital mortality (%)**	2(1%)	6(3.8%)	5(2.3%)	0.210
**Complication rate (%)**	67(33.5)	54(34.2%)	92(42.6%)	0.103
**Grade I**	20	13	19	
**Grade II**	23	19	40	
**Grade III**	14	11	18	
**Grade IIIa**	14	8	15	
**Grade IIIb**	0	3	3	
**Grade IV**	8	5	10	
**Grade IVa**	7	5	9	
**Grade IVb**	1	0	1	
**Grade V**	2	6	5	

**IQR:** interquartile range.

### Result of postoperative surgical complications, ICU stays, and hospital mortality

The postoperative data are shown in Table 4 and Table 5. The Clavien-Dindo Classification of surgical complications [26], [27] was used to evaluation the postoperative complications. The overall postoperative morbidity rate was 34.8% (200/574). However, there were no significant differences between three groups in postoperative complications: 33.5% in no occlusion group vs 34.2% in Pringle group vs 42.6% in hemi-hepatic group (P>0.05). The most common postoperative complications with grade III–V were bile leakage, pleural effusion, and liver failure. Only 1 patient in Pringle group and 1 in hemi-hepatic group need reoperation due to intra-abdominal hemorrhage.

**Table 5:** The main Types of complications.

	No occlusion group (n = 200)	The Pringle group (n = 158)	The hemi-hepatic group (n = 216)
**Bile leakage**	9	8	0
**Liver failure**	5	2	8
**Abdominal abscess**	1	5	1
**Lung infection**	15	12	17
**Wound infection**	5	2	6
**Pleural effusion**	4	5	6
**Intra-abdominal hemorrhage**	3	3	3

The overall hospital mortality was 2.3% (13/574) in this study: two in no occlusion group, six in Pringle group, and five in hemi-hepatic group. There were no significant differences between three groups in hospital mortality (P>0.05; 1% vs 3.8% vs 2.3% in no occlusion group, Pringle group, hemi-hepatic group, respectively). Among these patients, nine died of liver failure, two died of sudden cardiac arrest, and two died of perioperative intra-abdominal hemorrhage. The time of stay in the ICU did not reach significant differences between three groups (P>0.05).

## Discussion

Controlling blood loss in an effective, safe, and quick manner is the primary goal of liver surgeons when performing hepatectomy. The amount of intraoperative blood loss and perioperative transfusion requirements will markedly affect the postoperative liver function, postoperative morbidity and mortality, and long-term survival [8]–[10]. To decrease blood loss during hepatectomy, various hepatic vascular occlusion techniques have been developed such as total vascular exclusion [29], Pringle maneuver, hemi-hepatic vascular occlusion, hepatic vascular exclusion [30], and hepatic vascular exclusion with veno-venous bypass [31].

However, Pringle maneuver is most commonly used in clinical practice because of its simplicity, but it can induce ischemia-reperfusion injury to liver, which can result in metabolic, immunological, and microvascular changes [32]. Ischemia-reperfusion could even promote liver metastasis [15]. In addition, Pringle maneuver can induce visceral congestion and instability in hemodynamic status, and it can increase the mean arterial pressure by 10%, decrease in pulmonary artery pressure by 5% and cardiac index by 10% [33].

To avoid ischemia-reperfusion injury to the remnant liver and visceral congestion, some surgeons put forward ischemic preconditioning, which involves a brief period of ischemia and reperfusion to liver prior to Pringel vascular occlusion. However, the best time of ischemic preconditioning still remains to be study [34]–[36]. Because of this the technique of hemi-hepatic vascular inflow occlusion was suggested. It can block the blood supply of right or left hemi-hepatic portions at tumor location, allowing normal blood supply at the contralateral hemi-liver. However, one of concerns of the hemi-hepatic vascular occlusion is that there is a risk of bleeding from the contralateral hemi-liver. Liver surgeons should have experience in dissecting the hilar plate and lowering hilar plate to avoid injury to bile ducts and vessels.

Generally speaking, The Pringle group should have a higher postoperative serum albumin level. However, in this retrospective study, we found that there were no significant difference observed between three groups in bilirubin, liver enzymes, and albumin (P>0.05). There were two possible reasons: one was that intermittent vascular occlusion which could decrease ischemia-reperfusion injury to liver was applied when perform Pringle vascular occlusion; the other was that we use hooking with ligation to perform liver resection when Pringle maneuver was chosen. On the other hand, CUSA was used to perform liver parenchyma division when hemi-hepatic vascular occlusion or no vascular occlusion was selected. Hooking with ligation is a simple and quick method of liver resection, and the ischemia-reperfusion time of liver will be significantly reduced. On the contrary, the time of ischemia-reperfusion was prolonged when CUSA was used.

As all the patients were suffering from hepatocellular carcinomas with hepatitis B, most of them had postoperative hypoproteinemia. Ascites were the most common postoperative complications which usually required diuretic treatment or peritoneocentesis.

To the best of our knowledge, in a prospective random controlled study by Ni et al [37], it was reported that although there were no significance differences in blood loss between two groups such as Pringle maneuver and hemi-hepatic vascular occlusion, the complication rate after liver resection was higher in the Pringle maneuver group. Fu et al [38] reported that the two techniques were safe and efficacious, but the hemi-hepatic group had an earlier recovery of liver function than Pringle maneuver group. Chau et al [39] reported that hemi-hepatic group could respond better than Pringle maneuver group. When vascular occlusion time was ≥45 min, hemi-hepatic group had an earlier recovery of liver function. On the contrast, Tanaka et al [40] reported there were no differences between two groups in the perioperative data. Taniguchi et al [41] reported that though there were no significant difference in blood loss and operative time between Pringle maneuver group and no vascular occlusion group, and the liver enzymes and total bilirubin were elevated in Pringle group.

There are other known factors that affect the amount of blood loss and transfusion in the liver surgery. Various new surgical devices are used when performing hepatic resection such as water jet dissector, CUSA, and LigaSure (a vessel sealing system). A report by Une et al [42] indicated that although there were no differences in blood loss and operative time, water jet could show the surgical field more clearly. In our center, there is no strict standard for choosing the tools to perform hepatectomy. The choice is made according to the selection of vascular occlusion technique. Another known factor is the central venous pressure (CVP) during liver resection. A retrospective study by Smyrniotis reported that elevated CVP during major liver resections could result in greater blood loss and longer hospital stay [43]. However, a consensus was reached by liver surgeons that maintenance of a low CVP was always associated with less intraoperative blood loss and better postoperative outcomes. In our institution, a low central venous pressure in a range of 5 cm H_2_O is usually maintained.

At present, the two vascular occlusion techniques are selectively used in our center. When tumors limit within hemi-liver, hemi-hepatic vascular occlusion is usually chosen. If tumors are beyond the scope of hemi-liver, Pringle maneuver is preferred. If there is a massive hemorrhage in hepatic vein or injury to inferior vena cava, hepatic vascular exclusion is immediately applied.

In summary, this retrospective study showed that either Pringle maneuver or simple hemi-occlusion technique could be used safely and effectively to reduce complications during major hepatectomy. Although the overall complications rate between three groups did not reach significant differences, no vascular occlusion group had more blood loss than the other two groups during liver resection.
